# Inflammatory cells in the ascending aortic aneurysm in patients with type 2 diabetes versus patients with hypertension

**DOI:** 10.17305/bjbms.2021.6488

**Published:** 2021-10-12

**Authors:** Aleksandra Milutinović, Ruda Zorc-Pleskovič

**Affiliations:** 1 Institute of Histology and Embryology, Faculty of Medicine, University of Ljubljana, Ljubljana, Slovenia,; 2 International Center for Cardiovascular Diseases MC Medicor d.d., Izola, Slovenia

**Keywords:** Type 2 diabetes, arterial hypertension, ascending aortic aneurysm, inflammatory cells, pro-inflammatory macrophages, anti-inflammatory macrophages

## Abstract

Aortic aneurysms occur relatively frequently in the ascending thoracic aorta, but are rarely seen in patients with type 2 diabetes (DM2). Our aim was to evaluate inflammatory cell infiltration in the ascending aortic aneurysm wall in patients with diabetes without arterial hypertension (DM2 group, n = 6) versus hypertensive non-diabetic patients (AH group, n = 34). For histologic analysis, the sections were stained with hematoxylin-eosin and Movat pentachrome. The immunohistochemical staining was used to analyze the infiltration of pro-inflammatory (CD68) and anti-inflammatory macrophages (CD163), T helper (CD4) and T killer cells (CD8), and B (CD79a) and plasma cells (CD138) in all three layers of aneurysms of both groups. The statistical significance of the differences between groups was evaluated by ANOVA and the Welch test. In comparison to the AH group, the DM2 group developed less severe infiltration of pro-inflammatory macrophages (*p* = 0.004) and B cells (*p* = 0.025) in the tunica intima and tunica media (*p* = 0.049 and *p* = 0.007, respectively), and fewer plasma cells in the tunica media (*p* = 0.024) and tunica adventitia (*p* = 0.017). We found no significant differences in the number of T helper, T killer cells, and anti-inflammatory macrophages and in the amount of collagen and elastic fibers, ground substance, and smooth muscle cells in all three layers of the vessel wall. Except in tunica adventitia of DM2 group, there were more collagen fibers overall (*p* = 0.025). Thus, we conclude that the histological structure of the aneurysm in diabetics without hypertension is almost the same as in hypertensive patients without diabetes. Diabetics had significantly less inflammatory infiltration in all three layers of the vessel wall and more collagen fibers in tunica adventitia.

## INTRODUCTION

Type 2 diabetes (DM2) is characterized by chronic hyperglycemia caused by insulin deficiency due to pancreatic β-cell dysfunction and insulin resistance in target organs [1]. DM2 leads to microvascular and macrovascular complications. One of the major macrovascular complications is cardiovascular disease [1,2]. In contrast, experimental and epidemiologic studies showed a negative association between DM2 and the development of an aortic aneurysm, its expansion, and rupture [3].

The aneurysm is defined as local pathological widening of the artery diameter up to 1.5× [4,5]. Aortic aneurysms most commonly occur in the infrarenal region. The second most common location is the ascending thoracic aorta [6]. Aortic aneurysms usually expand asymptomatically until dissection or rupture of the aortic wall occurs, often leading to death [6].

The normal wall of ascending thoracic aorta differs from the abdominal aorta in structure and mechanical properties, cell biology, and biochemistry [6,7]. Compared to the abdominal aorta, ascending aorta has a thinner tunica intima, thicker tunica media, more medial lamellar units, higher collagen, elastin content, and a lower collagen-to-elastin ratio [6-8].

Furthermore, the ascending aortic aneurysm differs from the abdominal aortic aneurysm in the pathophysiological features. It has long been thought that the thoracic aortic aneurysm is characterized by a non-inflammatory medial degeneration known as medial cystic necrosis, while the abdominal aortic aneurysm is associated primarily with atherosclerosis and inflammation [4,5]. Subsequent studies, however, have shown that chronic inflammation is also present in the development of the thoracic aortic aneurysm, but less intensely [6,7,9,10]. Like the abdominal aortic aneurysm in the tunica intima, tunica media, and tunica adventitia of the thoracic aortic aneurysm, T cells (CD3) predominate among the inflammatory cells followed by macrophages (CD68). The B cells (CD20) were found in smallest amounts relative to other cells [6,9,11,12].

Risk factors such as male gender, arterial hypertension (AH), age, smoking, and dyslipidemia contribute to the development of the aortic aneurysm [3]. DM2, which is a risk factor for various micro- and macro-angiopathies, is not a risk factor for the development of aortic aneurysm. It was shown that aneurysms rarely develop in patients with DM2 [13,14].

Several studies from recent decades have shown that the pathogenesis of DM2 is associated with changes in the immune response. Insulin resistance and chronic hyperglycemia, which are characteristic of DM2, lead to chronic inflammation, endothelial dysfunction, increased advanced glycation end products (AGEs), cross-linking of collagen, and proliferation of vascular smooth vessel cells (VSMCs) [15]. DM2 also increases endothelial cell permeability, the expression of metalloproteinase-2 and -9 matrices, and the production of angiotensin 2 in vascular tissue [13]. The histopathological analysis of abdominal aortic aneurysms showed that DM2 patients had a higher macrophage infiltration compared to non-diabetics [16]. DM2 has been shown to modulate the macrophage phenotype and increase the ratio of pro- and anti-inflammatory macrophages [17]. However, glycation has been shown to modulate the macrophage phenotype against the anti-inflammatory state in the long term [17-19].

We hypothesized that the inflammatory infiltrates in the wall of the thoracic aortic aneurysm in patients with DM2 are different than in patients with AH. The purpose of the study is to analyze inflammatory/immune cell infiltration in the wall of the thoracic aortic aneurysm in patients with DM2 non-hypertensive compared with hypertensive non-diabetic patients. We wanted to evaluate the infiltration of macrophages (CD68 and CD163), T cells (CD4 and CD8), B (CD79a), and plasma cells (CD138) in tunica intima, tunica media, and tunica adventitia of the ascending aortic aneurysm.

## MATERIALS AND METHODS

### Patients

Ascending aortic aneurysms were obtained during the surgery. The study enrolled 40 patients with ascending aortic aneurysm with either DM2 only (DM2; n = 6) or AH only (AH group; n = 34). Exclusion criteria were AH and DM2 together, type 1 diabetes, and genetic mutations.

The study and all procedures were approved by the National Medical Ethics Committee (MEC 170/07/13, MEC 110/03/16). Written informed consent was obtained from each patient included in the study. The study protocol conforms to the ethical guidelines of the 1975 Declaration of Helsinki.

### Tissue samples

From specimens of ascending aorta that was ranging in length from 2 to 5 cm, the average three samples (from the bulge) were obtained, fixed in formalin for 24 hours, embedded in paraffin, and cut into 4.5 μm thick transversal step (step was 50 μm thick) serial sections. The sections were then stained with hematoxylin-eosin (HE) and Movat pentachrome [20]. The immunohistochemical staining was used to detect T killer – Tk cells (CD8; 1:50, Dako, Glostrup, Denmark), T helper – Th cells (CD4; 1:20, Cell Marque), B cells (CD79a; 1:20, Dako Glostrup, Denmark), plasma cells (CD138,1:30, Dako, Glostrup, Denmark), pro-inflammatory M1 macrophages (CD68;1:50, Dako, Glostrup, Denmark), and anti-inflammatory M2 macrophages (CD163; 1:40, Cell Marque) following the manufacturer’s instructions.

### Image analysis

Image analysis was performed using the computer program NIS elements (version 3 – documentation), a camera (Nikon digital sight DS-M5), and a light microscope (Nikon Eclipse E 400). The area of tunica intima, tunica media, and tunica adventitia was measured at an objective magnification of 4× and expressed in mm^2^.

The number (N) of CD4, CD8, CD79a, CD138, CD68, and CD163 positive cells in tunica intima, tunica media, and tunica adventitia was counted at the objective magnification of 40× in the whole section and expressed in N/mm2. The tunica adventitia was defined as a 500 μm wide band of connective tissue adjacent to the tunica media.

Movat pentachrome staining was used for stereological analysis with Weibel’s test system. The volume density of collagen fibers (yellow), mucin (blue-green), elastic fibers and nuclei (black), muscle (dark red), and fibrin (bright red color) in tunica intima, tunica media, and tunica adventitia was estimated as described previously [21].

### Statistical analysis

Analyses were performed using Microsoft Excel 2010 and Statistical Package for the Social Sciences SPSS 20. The average values of the measured parameters were calculated for DM2 and AH groups and expressed as the average value ± SD.

Due to an imbalance in the number of patients in the DM2 (n = 6) and AH groups (n = 34), the statistical significance of the differences between them was evaluated by ANOVA and the Welch test (*p* < 0.05) that is used to compare unequally sized samples.

## RESULTS

### Patients

Forty patients with ascending aortic aneurysms were divided into the DM2 group (n = 6) and AH group (n = 34). In both groups, there were more men than women. There were five women and 29 men in the AH group, and in the DM2 group, there were one woman and five men (Table 1). In the DM2 group, all patients were no smokers. In the AH group, five patients were smokers; the remaining 29 were no smokers (Table 1). Exclusion criteria were AH and DM2 together, genetic factors, and type 1 diabetes. There were no significant differences between groups in age, total cholesterol, low-density lipoprotein, high-density lipoprotein, triglyceride levels, creatinine, and urea in plasma (Table 1).

**TABLE 1:**
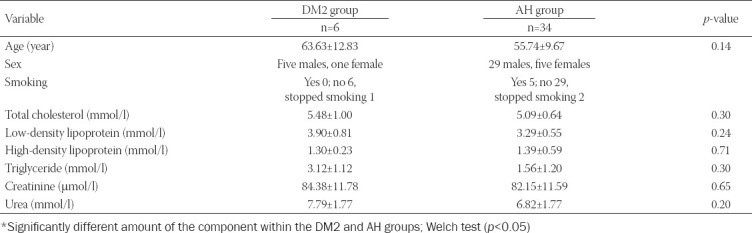
Characteristics for all patients in the AH and DM group (average±SD)

### Histological analysis

**FIGURE 1:**
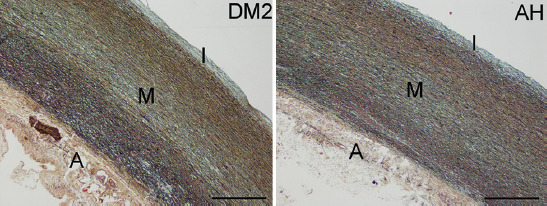
Tunica intima (I), tunica media (M), and tunica adventitia (A) of ascending aortic aneurysm in the DM2 and AH groups. Movat staining, bar = 600 μm, objective magnification of 4×.

The histological staining with HE and Movat (Figure 1) showed the tunica intima, tunica media, and tunica adventitia. Tunica intima was composed of endothelial cells that cover the subendothelial layer. Stereological analysis (Table 2) of the tunica intima of both groups showed that the most abundant was collagen, followed by elastic fibers, ground substance, and VSMCs. In tunica media of both groups, the most abundant is collagen, followed by VSMCs, elastic fibers, and a small amount of ground substance. The tunica adventitia of both groups was composed mostly of connective tissue with collagen fibers. There were very few elastic fibers, VSMCs, and ground substance.

**FIGURE 2:**
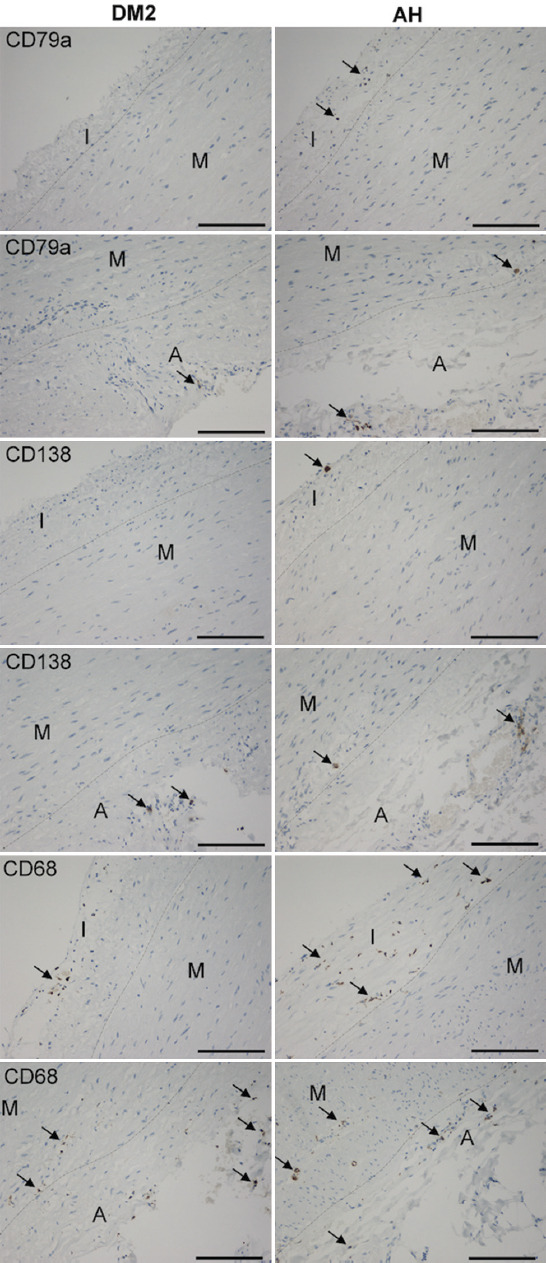
B cells (CD79a), plasma cells (CD138), and M1 macrophages (CD68) (arrows) in tunica intima (I), tunica media (M), and tunica adventitia (A) in the DM2 and AH groups. bar = 150 μm, objective magnification of 20×. The boundaries between the layers are shown by a dashed thin line.

**TABLE 2:**
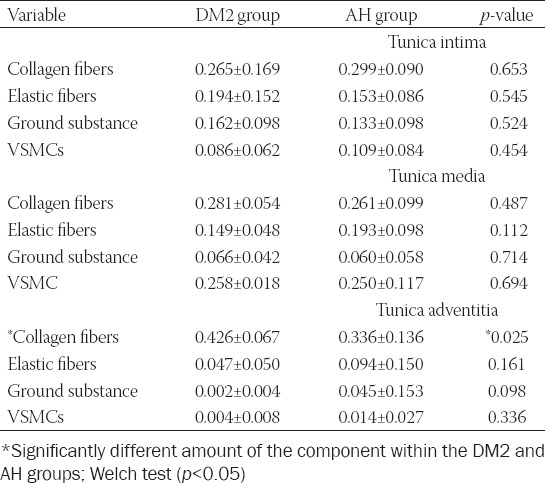
Stereological analysis of tunica intima, tunica media, and tunica adventitia of the samples of ascending aortic aneurysm in the DM2 and AH groups (average [mm/mm°]±SD)

Results of the stereological analysis are listed in Table 2. There were no significant differences in any component between DM2 and AH groups in tunica intima and tunica media. There was also no significant difference in the amount of elastic fibers, VSMCs, and ground substance in the tunica adventitia. However, in the tunica adventitia of the DM2 group were significantly more collagen fibers in comparison to the AH group.

### Comparison between AH and DM2 group in the number of inflammatory cells (N/mm2) within tunica intima, tunica media, and tunica adventitia

In most of our patients, positive inflammatory cells stained with CD79a (B cells), CD138 (plasma cells), CD68 (M1 macrophages), CD163 (M2 macrophages), CD4 (T helper – Th), and CD8 (T killer – Tk) were found in all three layers of the aneurysmal wall in at least one slice. In the tunica intima, tunica media, and tunica adventitia, the number of Th, Tk, B, and plasma cells, M1 and M2 macrophages were counted and expressed in N/mm2. The measurements are listed in Table 3. We found a smaller number of CD79a, CD138, and CD68 positive cells in DM2 than in the AH group in some layers of the arterial wall (Figure 2). In the DM2 group, there were significantly fewer B cells and M1 macrophages in tunica intima and tunica media than in the AH group. In the DM2 group, we also found significantly fewer plasma cells in tunica media and tunica adventitia than in the AH group. We found no significant differences in the number of M2 macrophages and T cells subtypes (CD4 and CD8) between the DM2 and AH groups.

**TABLE 3:**
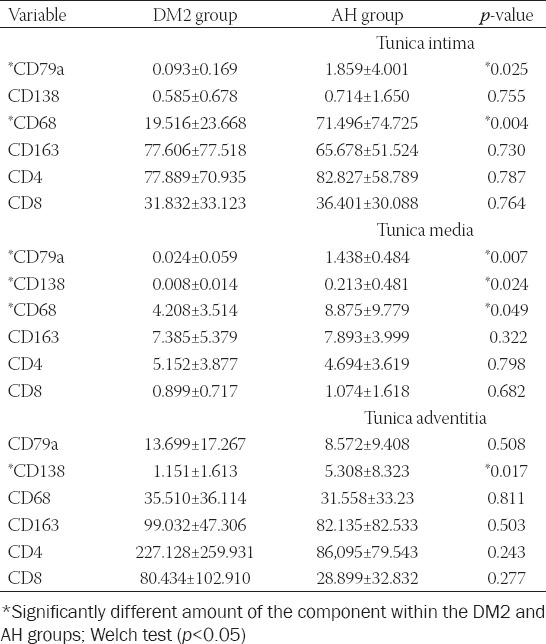
Analysis of inflammatory cells in the tunica intima, tunica media, and tunica adventitia of the ascending aortic aneurysm in the DM2 and AH groups (average N/mm^2^±SD)

## DISCUSSION

In this study, we investigated inflammatory/immune cell infiltration in the tunica intima, media, and adventitia of the ascending aortic aneurysm in patients with DM2 non-hypertensive compared with hypertensive non-diabetic patients. Compared with the AH group, patients with DM2 had fewer B cells and M1 macrophages in the tunica intima, fewer B cells, plasma cells, and M1 macrophages in the tunica media, and fewer plasma cells in the tunica adventitia. There were no differences in the number of M2 macrophages, Th, and Tk cells between both groups.

We also did not find significant differences in the amount of collagen and elastic fibers, VSMCs, the ground substance in all three layers of the aneurysmal wall between both groups, except in the tunica adventitia, where there were more collagen fibers in the DM2 group than in the AH group.

Our results indicate an inflammatory process in the wall of the ascending aortic aneurysm, as shown by numerous studies. In our study, inflammation was more severe in AH than in the DM2 group; more significant infiltration of M1 and B cells was in the intima and media, and more plasma cells in the media and adventitia of AH than DM2 group. Epidemiologic studies suggest that patients with DM2 have a lower incidence of the thoracic aortic aneurysm [22,23]. In the development of aneurysms, apoptosis, oxidative stress, inflammation, and enhanced proteolysis are involved [22]. They lead to loss of arterial wall matrix [5]. Contrary, for diabetes, the characteristic feature is excess and degeneration of matrix that leads to stiffness of the arterial wall [15,22,24]. It was shown that chronic hyperglycemia augments the production of AGEs, VSMCs proliferation, and collagen cross-linking, while insulin resistance enhances the expression of several genes involved the inflammatory processes and collagen synthesis [24,25]. It is known that compared with normal aortas, in aortic aneurysm, the amount of elastic and collagen fibers, glycosaminoglycans, and VSMCs is reduced [10]. In our study, we found no significant differences in the volume density of collagen and elastic fibers, ground substance, and VSMCs in the aneurysm wall between both groups. In the tunica adventitia alone, there were significantly more collagen fibers in the DM2 group than in the AH group.

It is known that diabetes modulates the inflammatory process. It enhances the production of inflammatory mediators that promote vascular inflammation [3] and, on the other hand, suppress it [26,27]. Studies in rats have shown that postprandial hyperglycemia (fluctuations in blood glucose) promotes monocyte adhesion to the thoracic aorta’s endothelium [3,28]. However, if they reduced blood glucose fluctuations, the adhesion of monocytes to endothelial cells was reduced [3,28]. Contrary, it was shown that hyperglycemia inhibits macrophage activation through the activation of liver X receptor β, but not it’s migratory ability [5]. Increased recruitment of monocytes leads to the development of an aneurysm only if the monocytes differentiate into pro-inflammatory macrophages [3,29]. However, hyperglycemia by different mechanisms modulates the macrophage phenotype into either the pro- [30,31] or anti-inflammatory type [17]. M1 macrophages can also blunt the inflammatory response [32]. In a model of myocardial infarction in mice, it was found that early pro-inflammatory macrophages secrete large amounts of matrix metalloproteinase-9 [32,33]. Matrix metalloproteinase-9 can mitigate the inflammatory response by cleaving the receptor for AGEs into soluble form, which has anti-inflammatory properties [32,34]. Pro-inflammatory macrophages have been reported to predominate after short-term diabetes and in association with hypercholesterolemia [30], while anti-inflammatory M2 macrophages predominate after diabetes lasting months [17]. In our study, where diabetes lasted for several years, the DM2 group had fewer M1 macrophages in the tunica intima and tunica media, but not in tunica adventitia, compared to the AH group, while the groups did not differ in the number of M2 macrophages. It was also shown that patients with DM2 had more CD68-positive macrophages in abdominal aortic aneurysm than non-diabetic patients and no difference in the number of T cells [3,16]. However, contrary to humans, studies on mice models of abdominal aortic aneurysm revealed a decrease of macrophage infiltration in diabetic compared to non-diabetic mice [3,26,35,36]. Our results are more similar to studies in a mouse model [3,26,35,36] than studies in humans [3,16], as diabetic patients in our study had less inflammatory infiltration of M1 and B cells in the tunica intima and tunica media, and fewer plasma cells in the tunica media and tunica adventitia. Differences in results could be attributed to selected groups regarding hypertension and diabetes. In a human study, 77% of patients in the diabetes group also had hypertension and 72% in the non-diabetes group [16]. In animal studies, groups of mice with diabetes did not have hypertension [26,35,36]. Therefore, groups of mice with diabetes, and consequently the results of the study, are more comparable to the DM2 group in our study than to the diabetic group in human studies [16].

Patients with DM2 without AH who develop the ascending aortic aneurysm are known to be relatively rare [37,38]. The latter is the reason our DM2 group was small. The small number of participants in the DM2 group is, however, the main limitation of our study.

In our study, we found no differences in the number of T cells between the AH and DM2 groups, which are consistent with the observations of other researchers [3,16]. We also found no differences between the AH and DM2 groups in infiltration with M2 macrophages. Compared with the AH group, patients with DM2 in our study had fewer B cells in the tunica intima, fewer B cells and plasma cells in the tunica media, and fewer plasma cells in the tunica adventitia. The experimental model of abdominal aortic aneurysm revealed the protective role of B cells and plasma cells depletion against aneurysmal formation [10,39].

The prevalence of aneurysms in patients with diabetes without hypertension is low [37,38]; diabetes obviously protects them from developing an aneurysm. Patients in our DM2 group developed the aneurysm, although they had significantly fewer B and plasma cells and fewer pro-inflammatory macrophages, that is, cells that are otherwise involved in aneurysm development. There were no significant differences in the infiltration of Th, Tk, and M2 cells and in the amount of collagen and elastic fibers, ground substance, and VSMCs in all three layers of the vessel wall. Except in the adventitia of DM2 group, there were more collagen fibers.

## CONCLUSION

Aneurysms of ascending aorta are known to occur less frequently in diabetics than in hypertensive patients. We conclude that the histological structure of the aneurysm in diabetics without hypertension is almost the same as in hypertensive patients without diabetes. Diabetics had significantly more collagen fibers in the tunica adventitia and significantly less inflammatory infiltration in all three layers of the vessel wall.
